# Degradation of specific glycosaminoglycans improves transfection efficiency and vector production in transient lentiviral vector manufacturing processes

**DOI:** 10.3389/fbioe.2024.1409203

**Published:** 2024-06-26

**Authors:** Thomas Williams-Fegredo, Lee Davies, Carol Knevelman, Kyriacos Mitrophanous, James Miskin, Qasim A. Rafiq

**Affiliations:** ^1^ Oxford Biomedica (UK) Limited, Oxford, United Kingdom; ^2^ Department of Biochemical Engineering, Advanced Centre for Biochemical Engineering, University College London, London, United Kingdom

**Keywords:** lentiviral vector, glycosaminoglycan, chondroitin sulphate, chondroitinase ABC, transient transfection, transient gene expression, gene therapy

## Abstract

Both cell surface and soluble extracellular glycosaminoglycans have been shown to interfere with the exogenous nucleic acid delivery efficiency of non-viral gene delivery, including lipoplex and polyplex-mediated transfection. Most gene therapy viral vectors used commercially and in clinical trials are currently manufactured using transient transfection-based bioprocesses. The growing demand for viral vector products, coupled with a global shortage in production capability, requires improved transfection technologies and processes to maximise process efficiency and productivity. Soluble extracellular glycosaminoglycans were found to accumulate in the conditioned cell culture medium of suspension adapted HEK293T cell cultures, compromising transfection performance and lentiviral vector production. The enzymatic degradation of specific, chondroitin sulphate-based, glycosaminoglycans with chondroitinase ABC was found to significantly enhance transfection performance. Additionally, we report significant improvements in functional lentiviral vector titre when cultivating cells at higher cell densities than those utilised in a control lentiviral vector bioprocess; an improvement that was further enhanced when cultures were supplemented with chondroitinase ABC prior to transfection. A 71.2% increase in functional lentiviral vector titre was calculated when doubling the cell density prior to transfection compared to the existing process and treatment of the high-density cell cultures with 0.1 U/mL chondroitinase ABC resulted in a further 18.6% increase in titre, presenting a method that can effectively enhance transfection performance.

## Introduction

Lentiviral vectors (LVVs), derived from the human immunodeficiency virus type-1 (HIV-1), are widely used gene therapy delivery vehicles employed to carry therapeutic transgenes into target tissues and organs for the treatment of debilitating acquired and inherited diseases (Bulcha et al., 2021). LVVs have a number of properties that make them ideal gene therapy vectors including their ability to 1) effectively transduce dividing and non-dividing cells, 2) their low immunogenicity, 3) their high packaging capacity, and 4) their ability to integrate genetic material into the host cell genome resulting in long term transgene expression (Mátrai et al., 2010). At the present time, gene therapy viral vectors used both commercially and in clinical trials are predominantly manufactured via transient transfection-based bioprocesses (van der Loo and Wright, 2016). The FDA anticipate the approval of between 10–20 new cell and gene therapy products a year by 2025 (Gottlieb, 2019). With an increasing number of therapies reaching late-stage clinical trials and being commercialised, it is evident that demand for viral vector products will continue to exceed existing global production capabilities unless significant technology improvements emerge and production capacity increases (Gottlieb, 2019; Masri et al., 2019).

Synthetic cationic lipids and cationic polymers represent categories of transfection reagents which form structures known as lipoplexes and polyplexes, respectively, when complexed with nucleic acid. These are extensively employed across the sector to facilitate exogenous gene transfer due to their effectiveness *in vitro* and limited cytotoxicity (Luo and Saltzman, 2000; Kim and Eberwine, 2010; Ma et al., 2021). To ensure a successful transfection via nucleic acid delivery, lipoplexes and polyplexes must pass their contents across the plasma membrane. The initial binding of cationic lipoplexes and polyplexes to the negatively charged cell surface is believed to be mainly mediated by electrostatic interactions (Elouahabi and Ruysschaert, 2005).

Glycosaminoglycans (GAGs) are large, linear, heterogeneous polysaccharides consisting of a series of repeating disaccharide subunits which have been shown to alter the delivery efficiency of cationic based gene delivery systems, including lipoplexes and polyplexes (Mislick and Baldeschwieler, 1996; Ruponen et al., 2004; Nomani et al., 2014). Glycosaminoglycans are ubiquitously expressed and are abundant on both the surface of cells and in the extracellular matrix; they are frequently covalently attached to core proteins, forming proteoglycans, but can also exist as soluble molecules. There are five types of glycosaminoglycans: hyaluronan, chondroitin, dermatan, heparin/heparan, and keratan (Couchman and Pataki, 2012; Sodhi and Panitch, 2020). With the exception of hyaluronan, all types of GAGs are sulphated giving the chains a negative charge (Gulati and Poluri, 2016).

**FIGURE 1:**
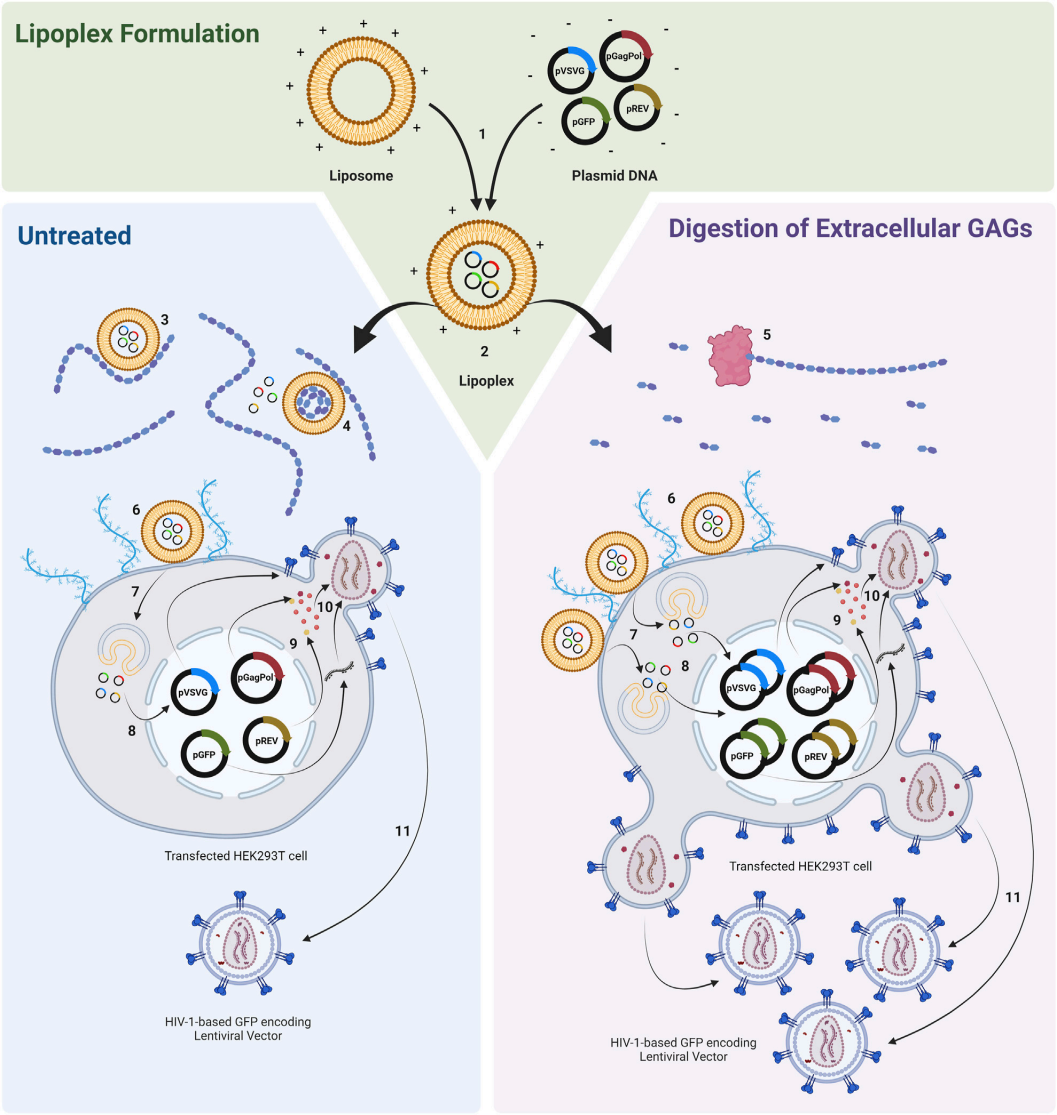
Transfection of HEK293T cell populations with lipoplexes complexed with plasmids required to produce a HIV-1-GFP based LVV. Cell populations are either untreated or treated with GAG digesting enzymes prior to transfection. 1) *Ex situ* complexion of liposomes and plasmid DNA. 2) Addition of lipoplexes to cell culture. 3) Interaction of anionic GAGs with cationic lipoplexes may modify the electrochemical and physiochemical properties of complexes and may potentially compete with cell surface proteoglycans for complex binding, impeding the ability of lipoplexes to localise and bind to the negatively charged cell membrane. 4) Binding of GAGs to lipoplexes can release nucleic acid from complexes. 5) Enzymatic degradation of extracellular GAGs. 6) Binding of lipoplexes to cell membrane. 7) Endocytosis. 8) DNA release and nuclear entry (complexes associated with bound GAGs may be subjected to altered endocytic routes and may exhibit altered, abnormal, behaviour within the endosome). 9) Plasmid expression. 10) Vector assembly. 11) Vector budding. Figure made using BioRender.

Whilst cell surface GAGs have been shown to serve as the primary receptors for many viruses, their role in mediating non-viral gene delivery, including lipoplex and polyplex-based transfection, is ambiguous and somewhat contradictory (Mislick and Baldeschwieler, 1996; Mounkes et al., 1998; Ruponen et al., 2004; Payne et al., 2007; Naik et al., 2011). The impact of soluble glycosaminoglycans on transfection, however, is less ambiguous and the exogenous addition of GAGs has been shown to decrease the efficiency of gene delivery during *in vitro* transfection studies. Sulphated GAGs have both a comparable size and charge to DNA and have been shown to interact with cationic lipids (Ruponen et al., 1999). The addition of heparin and heparan sulphate as well as the synthetic glycosaminoglycan analogue, dextran sulphate, to cell culture medium has been shown to block lipoplex mediated transfection (Mislick and Baldeschwieler, 1996). Soluble glycosaminoglycans may interfere with lipoplex and polyplex-mediated transfection in several ways (Figure 1). The binding of anionic GAGs to cationic lipoplexes and polyplexes may modify the size and charge of such complexes and may potentially compete with cell surface GAGs for complex binding, impeding the ability of lipolexes and polyplexes to localise and bind to the negatively charged cell membrane, decreasing cellular uptake (Tyagi et al., 2001). The extracellular binding of GAGs has also been shown to release nucleic acid from complexes, compromising the ability of the complex to deliver the transgene to target cells (Zelphati et al., 1996; Ruponen et al., 1999; Burke and Pun, 2008). Furthermore, it has been proposed that lipoplexes and polyplexes associated with bound GAGs may be subjected to altered endocytic routes and may exhibit altered, abnormal, behaviour within the endosome. This may compromise the ability of the nucleic acid to escape into the cytosol and alter its intracellular distribution (Ruponen et al., 2001). It is recognised, therefore, that GAG accumulation in conditioned cell culture medium over time may present a considerable barrier to transfection.

There is an absence of work conducted to specifically remove soluble GAGs from conditioned medium prior to transfection with the aim of increasing transfection efficiency and recombinant protein expression. This may be achieved via the treatment of cultures with specific GAG digesting enzymes which is hypothesised to increase both transfection performance and LVV production as illustrated in Figure 1. Alternatively, GAGs may also be removed from cultures by employing a less targeted, medium exchange step prior to transfection which involves the removal of conditioned medium followed by the replacement with fresh medium. However, medium exchanges are challenging to perform in the context of large-scale suspension manufacturing processes and the development of commercial cell culture media which are compatible with cell growth, transfection and LVV production (such as Freestyle 293) have meant that pre-transfection medium exchanges are not routinely performed in suspension LVV bioprocesses. A targeted, enzymatic, degradation may therefore be more beneficial in applications where the removal of GAGs via medium replacement strategies is not practical or is complex to scale-up, or in applications where the retention of certain critical components in conditioned medium, including various growth factors, metabolites and signalling molecules, may be important. This study assessed the impact of various soluble glycosaminoglycans and anionic polysaccharides on transfection performance and determined whether the degradation of specific glycosaminoglycans could be used to enhance the performance of a lentiviral vector production bioprocess. Three metrics were used to assess transfection performance:

1) Transfection efficiency: the percentage of cells in a transfected population that express the transgene encoded in the transfer plasmid.
2) GFP production: to measure the relative level of transgene expression in a transfected cell population.
3) Functional LVV titre: as determined by the transduction of adherent HEK293T cells with HIV-1-based LVV particles produced from the transfected cells.

## Materials and methods

### Chemicals

Chondroitin sulphate (derived from shark cartilage), dextran (molecular mass: 6,000, *Leuconostoc* spp.), dextran sulphate sodium salt (molecular mass: 5,000, *Leuconostoc* spp.), heparin sodium salt (derived from porcine intestinal mucosa), dextranase (*Penicillium* sp.), chondroitinase ABC (*Proteus vulgaris*), heparinase I/III blend (*Flavobacterium heparinum*) were purchased from Sigma Aldrich (Sigma-Aldrich, Merck, Burlington, MA, United States).

### Cell culture

Suspension-adapted HEK293T cells, provided by Oxford Biomedica, were routinely passaged (sub-cultured) in, serum-free, FreeStyle 293 Expression Medium (Gibco, Thermo Fisher Scientific, Waltham, MA, United States) and maintained in a shaking incubator (orbital shaking diameter of 25 mm) at 37°C, 300 rpm, 5% CO_2_. Cells were cultivated in 24-deep well plates (24-DWPs) and Erlenmeyer shake flasks, at working volumes of 3 mL and 25 mL, respectively.

### Lentiviral vector production

Recombinant, pseudotyped, replication incompetent, lentiviral vectors (LVVs) were produced using Oxford Biomedica’s propriety LentiVector^®^ delivery platform. HIV-1-based LVVs were produced via the transient co-transfection of suspension-adapted HEK293T cells with third-generation packaging plasmids. Cells were cultured for approximately 24 h prior to transfection. Four plasmids were co-transfected in total consisting of a vector genome transfer plasmid encoding green fluorescent protein (pOXB-GFP), two separate packaging plasmids: one encoding Rev (pOXB-REV) and one encoding Gag and Pol (pOXB-HSGP) and a plasmid encoding the vesicular stomatitis virus G (VSV-G) envelope protein (pOXB-VSV-G). Cells were transfected with lipoplexes, prepared via the combination of the plasmids complexed with the cationic lipid, Lipofectamine™ 2000CD (Invitrogen, Thermo Fisher Scientific, Waltham, MA, United States) according to the manufacturer’s guidelines and under ambient conditions. A total of 1.2 µg/mL of total plasmid DNA was added to cultures and a Lipofectamine™ 2000CD: total DNA mass ratio of 4:1 was used. Cultures were transfected at cell densities of 2 × 10^6^ viable cells/mL for all experiments except for the high cell density condition where cultures were transfected at cell densities of 4 × 10^6^ viable cells/mL. Transfected cell populations were supplemented with sodium butyrate (Sigma-Aldrich, Merck, Burlington, MA, United States), to achieve a final concentration of 10 mM. The HIV-1-based LVV containing supernatant was isolated approximately 48 h post-transfection, clarified through a 0.45 µm filter and stored at −80°C for subsequent analysis.

### Transfection efficiency analysis

HEK293T cells were removed from vector production cultures approximately 24 h post-transfection and populations analysed with a 488 nm excitation laser using an Attune NxT acoustic focusing flow cytometer (Thermo Fisher Scientific, Waltham, MA, United States). Analysis was terminated when 10,000 live cell events had been processed. Subsequent data analysis was performed using FlowJo (FlowJo LLC, Ashland, OR, United States) and transfection efficiencies determined using Eq. 1 (Supplementary Figure S1). The reported GFP production metric was a measure of the intensity of GFP expression for the entire live cell population and was calculated by multiplying the median fluorescence intensity (MFI) of the gated transgene positive cell population by the percentage of transgene positive cells (Eq. 2). The GFP production metric was used as a measure of the relative level of transgene expression between different cell populations. This method has been used previously by others as a more accurate way to express GFP production; higher MFI values indicate higher GFP production in transgene positive cells but not necessarily a higher level of GFP expression for the entire cell population (Ruiz de Garibay et al., 2013).

### Functional vector titre

Functional lentiviral vector titre was determined via the transduction of adherent HEK293T cells at a density of approximately 2 × 10^5^ viable cells with HIV-1-based LVV particles in a 1,500 µL volume. LVV particle preparations were serially diluted, 400-fold, in DMEM (Sigma-Aldrich, Merck, Burlington, MA, United States) supplemented with 8 µg/mL polybrene (Sigma-Aldrich, Merck, Burlington, MA, United States). Two replicate wells of adherent cells were transduced with each sample analysed and the medium was not changed after transduction. Transduced cells were harvested 72 h following their exposure to the diluted viral vector preparations and subjected to analysis via flow cytometry using an Attune NxT acoustic focusing flow cytometer. The total number of single, live, transgene positive cells as a percentage of the total number of single, live cells was determined via analysis of the cell populations on FlowJo. Assays were deemed valid if transduced cell populations exhibited a total percentage of GFP positive cells of less than 30% for each sample. Eq. 3 was used to determine the concentration of functional transducing units/mL.

### Quantification of sulphated glycosaminoglycans

Conditioned cell culture medium containing soluble extracellular glycosaminoglycans was isolated from HEK293T cells via centrifugation. The metachromatic dye, 1, 9-dimethylmethylene blue, was used to quantify the concentration of sulphated glycosaminoglycans in test samples using a “Sulphated Glycosaminoglycan Detection Kit” (AMS Biotechnology, Abingdon, Oxford, United Kingdom), according to the manufacturer’s protocol. The dye does not discriminate between different sulphated glycosaminoglycans and detects chondroitin-4-sulphate, chondroitin-6-sulphate, dermatan sulphate, heparin sulphate and keratan sulphate. 100 µL of dye was added to 100 µL of each sample in microwell plates and the resulting shift in the absorption spectrum read using a SpectraMax^®^ i3x spectrophotometer (Molecular Devices LLC, San Jose, CA, United States) at 525 nm.

### Statistical analysis

Differences between means were evaluated using one-way ANOVA followed by Dunnett *post hoc* multiple comparison tests. Statistical tests were performed using GraphPad Prism V9.1 (GraphPad Software, San Diego, CA, United States) and values considered to be statistically significant when *p*-values were less than 0.05 (*), 0.01 (**), 0.001 (***), 0.0001 (****). Where the number of replicates (*n*) is quoted, this refers to independent biological replicates in every case.

### Diagrams and schematics

Diagrams and schematics were made using BioRender.com (BioRender, Toronto, Canada). All graphs were produced using GraphPad Prism V9.1.

### Equations

Transfection Efficiency

$$Transfection\;Efficiency\;\left(\%\right)=\left(\frac{Gated\;single,live,transgene\;positive\;cells}{Gated\;single,live\;cells}\right)\times 100$$ (1)

GFP Production

$$GFP\;Production=MFI\;of\;gated\;single,live,trangene\;positive\;cells\times \frac{Transfection\;Efficiency\;\left(\%\right)}{100}$$ (2)

Functional Vector Titre

$$Titre\;\left(TU/mL\right)=\frac{\left[\frac{\%\;of\;transgene\;positive\;cells\;}{100}\times Number\;of\;cells\;prior\;to\;transduction\times Dilution\;factor\right]}{Volume\;of\;vector\;added\;\left(mL\right)}$$ (3)

## Results and discussion

### Glycosaminoglycans accumulate in conditioned cell culture medium of suspension adapted HEK293T cells

**FIGURE 2:**
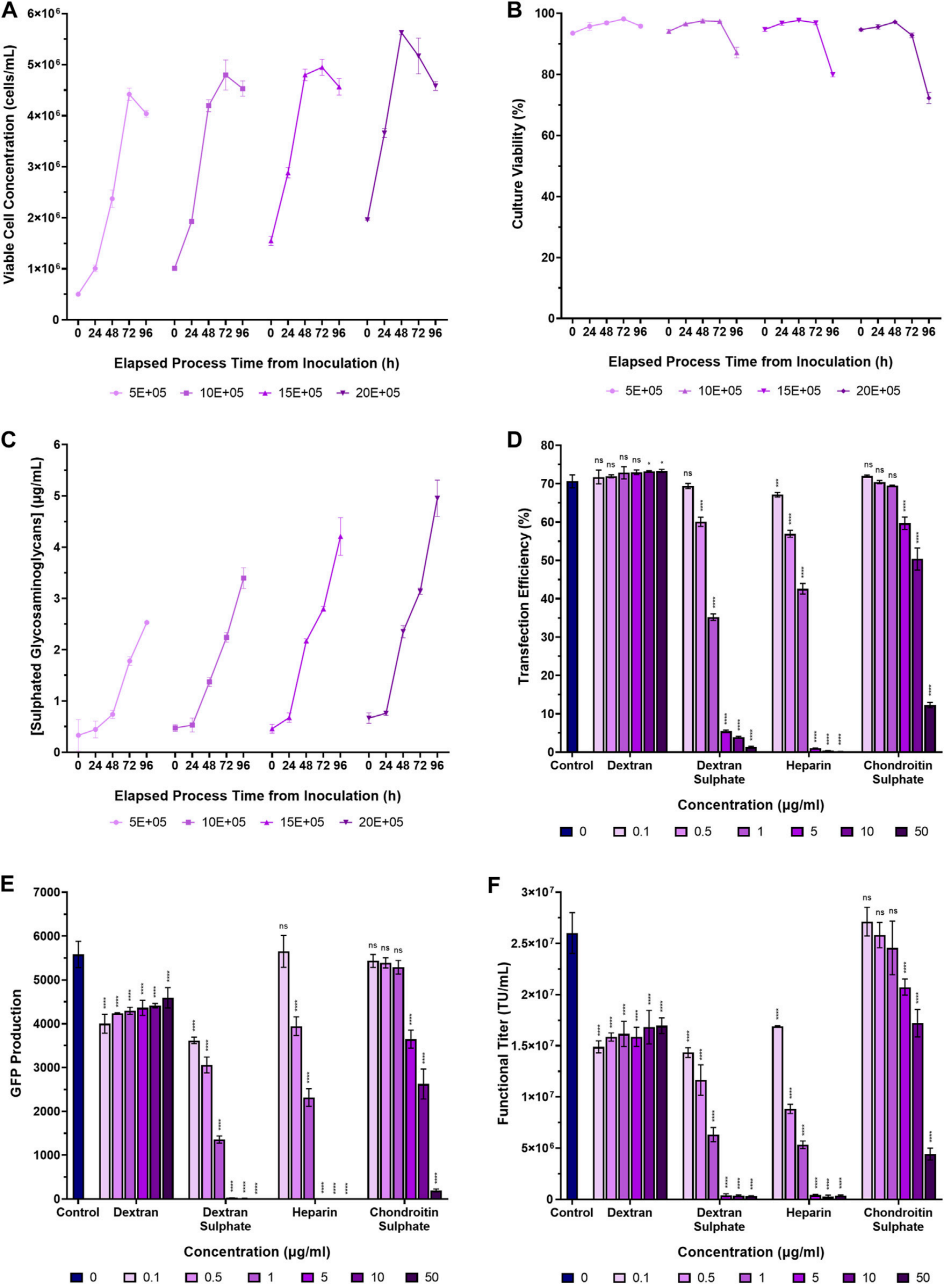
Accumulation of sulphated glycosaminoglycans in conditioned medium and transfection performance when supplementing cultures with various glycosaminoglycans and synthetic GAG-analogues. **(A)** Viable cell concentration, **(B)** culture viability, and **(C)** concentrations of extracellular sulphated glycosaminoglycans in suspension adapted HEK293T cultures over a 96-h incubation period. **(D)** Transfection efficiency, and **(E)** GFP production data measured 24 h post-transfection following the supplementation of HEK293T cultures with dextran, dextran sulphate, heparin and chondroitin sulphate, 4 h pre-transfection. **(F)** Functional LVV titres achieved under the different conditions. The “Control” in panels **(D–F)** corresponds to the addition of no GAGs. Data points **(A–F)** are the average of triplicates with the exception of data points labelled as “control” in **(D–F)** which are the average of 12 replicates. All error bars are ± one standard deviation of the mean.

Glycosaminoglycans, produced by almost all animal cells, are secreted into the dynamic and complex environment of the extracellular matrix in adherent cell systems where they have roles in defining tissue architecture and regulating vital cellular processes and functions (Soares Da Costa et al., 2017; Silva et al., 2019). To first examine whether suspension adapted HEK293T cells secreted glycosaminoglycans into the extracellular environment, Erlenmeyer 125 mL shake flasks were inoculated at four cell densities ranging from a concentration of 5.0 × 10^5^ viable cells/mL to 2.0 × 10^6^ viable cells/mL and incubated for 96 h. Supernatant samples were acquired daily to facilitate the quantification of extracellular glycosaminoglycans in the conditioned medium over time. Cells were initially centrifuged, washed and resuspended in fresh FreeStyle 293 Expression Medium to minimise the transfer of residual extracellular glycosaminoglycans into the cultures from the previous sub-culture. Sulphated GAGs were found to accumulate in conditioned medium over time, increasing both with higher cell concentrations and longer incubation times (Figures 2A–C). Final extracellular sulphated GAG concentrations of 2.5 µg/mL, 3.4 µg/mL, 4.2 µg/mL, and 5.0 µg/mL were measured in suspension cultures inoculated at densities of 5.0 × 10^5^ viable cells/mL, 1.0 × 10^6^ viable cells/mL, 1.5 × 10^6^ viable cells/mL, and 2.0 × 10^6^ viable cells/mL, respectively, following a 96-h incubation period. Since soluble extracellular glycosaminoglycans are widely considered to be inhibitory to non-viral mediated gene delivery systems, the accumulation of such biomolecules, as exhibited in this experiment, may present a significant biological barrier to a transient transfection unit operation. Upon completion, we next sought to quantify the impact of extracellular glycosaminoglycans on transfection performance.

### Addition of glycosaminoglycans and the GAG analogue, dextran sulphate, compromises transfection performance

To assess the impact of certain glycosaminoglycans and sulphated polysaccharides on transfection performance and LVV production, suspension-adapted HEK293T cultures in 24-DWPs were supplemented with dextran, dextran sulphate, heparin and chondroitin sulphate between concentrations of 0.1 µg/mL to 50 µg/mL, 4 h prior to transfection with plasmids required to produce a HIV-1-GFP LVV.

Addition of dextran to cultures prior to transfection had a minimal impact on transfection efficiency at the concentrations assessed but was found to significantly reduce both GFP production (*p* < 0.0001) and functional LVV titre (*p* < 0.0001), even at the lowest concentration that was evaluated (Figures 2D–F). The GAG analogue, dextran sulphate, was observed to be a potent inhibitor of transfection with a significant reduction in transfection efficiency being observed at a concentration of 0.5 µg/mL (*p* < 0.0001). The increased potency of the inhibitory effect exhibited by dextran sulphate is likely attributable to the higher degree of sulphation, and resulting high negative charge density, compared to dextran. In support of this, the sulphur content of the chondroitin sulphate and dextran sulphate used in this experiment was 4% and 19%, respectively, and chondroitin sulphate was observed to have a reduced inhibitory effect on transfection performance compared to dextran sulphate. A concentration of 5 µg/mL chondroitin sulphate was required to achieve the equivalent inhibitory effect on transfection efficiency and GFP production of dextran sulphate at a concentration of 0.5 µg/mL. Whilst the sulphur content of the heparin used in this experiment was not tested by the supplier, heparin from porcine intestinal mucosa is reported to have a sulphur content of 11.3%–12.4% (Aviezer et al., 1994; Landau et al., 2001; Ziegler and Seelig, 2008; Guarino et al., 2021). The overall sulphation density of the species investigated was therefore: dextran sulphate > heparin > chondroitin sulphate > dextran and the sulphation density was found to be directly proportional to the potency of inhibition on transfection performance.

The notion that more highly sulphated biomolecules have an increased inhibitory effect on transfection has been demonstrated previously. Chondroitin sulphate B, which is more highly sulphated that chondroitin sulphates A and C, was previously shown to reduce luciferase expression to a greater degree when the chondroitins were individually added to HeLa cells prior to transfection (Mislick and Baldeschwieler, 1996). Similarly, in addition to influencing non-viral mediated gene delivery, the degree of glycosaminoglycan sulphation has also been reported to influence viral mediated gene delivery systems. Le Doux et al., 1998 demonstrated that the inhibition of viral transduction was dependent on the degree of sulphation, reporting that chondroitin sulphate A was 100-fold more potent than the un-sulphated GAG, hyaluronic acid, in inhibiting viral transduction (Doux et al., 1999). Overall, the results reported in our study are in agreement with earlier work which previously demonstrated that anionic polysaccharides are potent inhibitors of polycation mediated transfection (Mislick and Baldeschwieler, 1996; Ruponen et al., 2001).

### Chondroitinase ABC enhances transfection performance compared to other GAG-digesting enzymes

**FIGURE 3:**
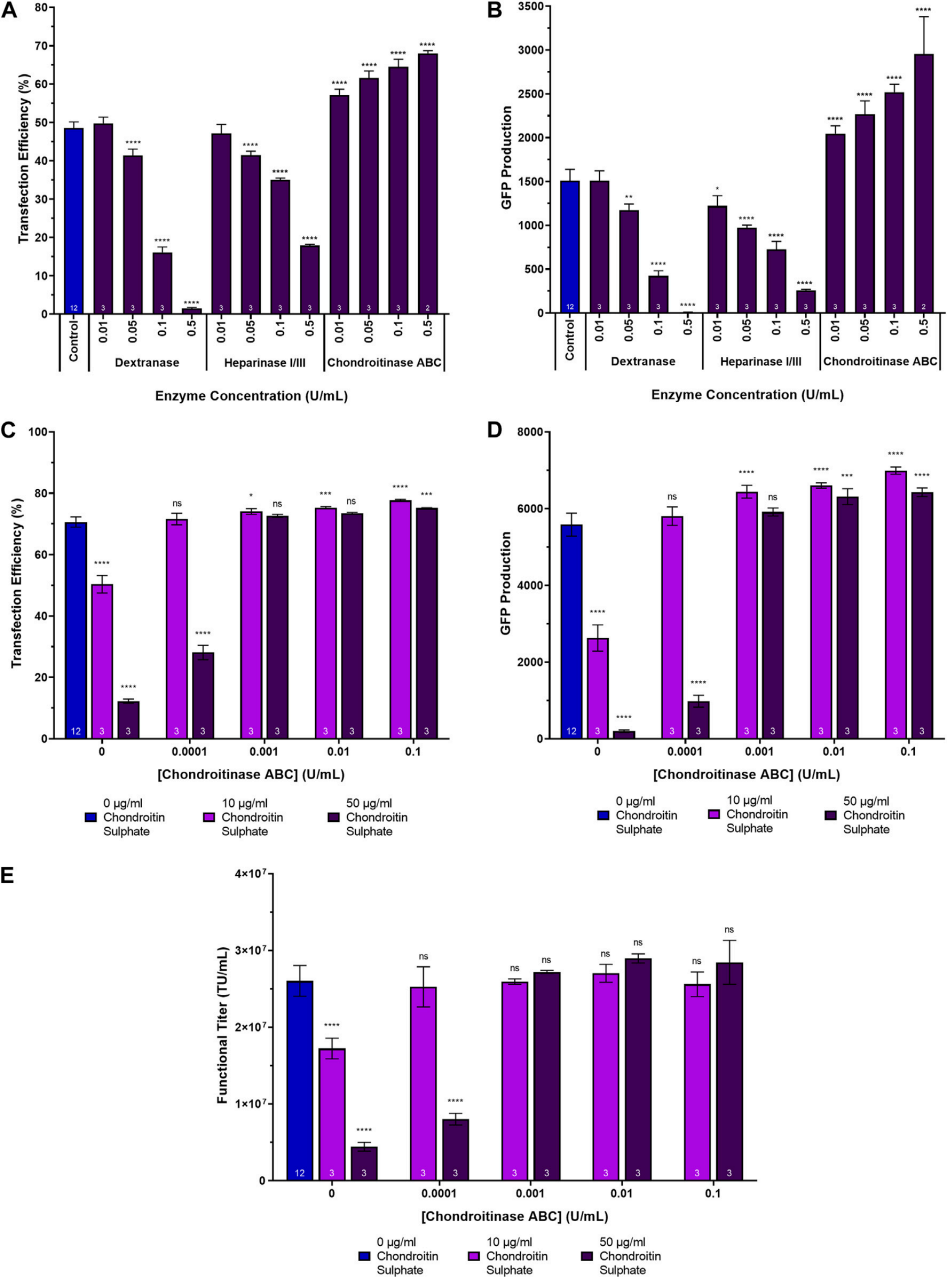
Transfection performance when supplementing cultures with dextranase, heparinase I/III and chondroitinase ABC. **(A)** Transfection efficiency and **(B)** GFP production data measured 24 h post-transfection following the supplementation of HEK293T cultures with dextranase, heparinase I/III and chondroitinase ABC, 2 h pre-transfection. **(C)** Transfection efficiency, **(D)** GFP production data measured 24 h post-transfection, and **(E)** functional LVV titres achieved following the supplementation of HEK293T cultures with varying concentrations of chondroitin sulphate 4 h prior to transfection and varying concentrations of chondroitinase ABC 2 h following the chondroitin sulphate additions. Data points **(A–E)** are the average of triplicates with the exception of data points labelled as “control” which are the average of 12 replicates. All error bars are ± one standard deviation of the mean.

To assess the impact of degrading glycosaminoglycans and sulphated polysaccharides present in the conditioned culture medium on transfection performance, suspension adapted HEK293T cultures in 24-DWPs were supplemented with dextranase, a heparinase I/III blend and chondroitinase ABC between a concentration range of 0.01 U/mL to 0.5 U/mL for each species. The enzymes were added 2 h prior to transfection with plasmids encoding a HIV-1-GFP LVV. Chondroitinase ABC was found to be the most promising candidate to enhance transfection performance; it significantly increased transfection efficiency and GFP production at all concentrations tested, in a dose dependent manner. The highest chondroitinase ABC concentration of 0.5 U/mL resulting in a 19.4% increase in transfection efficiency, corresponding to a 2.0-fold increase in GFP production compared to the untreated control (Figures 3A, B). Conversely, the addition of both dextranase and heparinase I/III were found to be detrimental to transfection, reducing transfection efficiency by 47.1% and 30.7%, corresponding to a 200-fold and a 6-fold reduction in GFP production, respectively, at the highest concentrations evaluated. This result could potentially be attributable to the enzymatic cleavage and degradation of key cell surface proteoglycans, including heparin/heparan sulphate proteoglycans, which may be important for mediating the initial electrostatic interaction and binding of lipoplexes to the cell membrane (Labat-Moleur et al., 1996). Indeed, transfection efficiency was significantly reduced in a proteoglycan-deficient Chinese hamster ovary (PGD-CHO) cell line with both polyethylenimine and Lipofectamine-mediated transfection compared to wild-type CHO cells (Mislick and Baldeschwieler, 1996; Payne et al., 2007) and Raji cells, which do not express proteoglycans, could not be transfected with lipoplexes until they were induced to express the proteoglycan, syndecan-1 (Mounkes et al., 1998). Interestingly, whilst Mislick and Baldeschwieler previously reported a 78% reduction in luciferase expression following treatment of HeLa cells with heparinase which was concordant with our findings, they also reported a 20% decrease in luciferase expression following treatment with chondroitinase ABC, relative to controls, which is contrary to our observations (Mislick and Baldeschwieler, 1996). However, it should be noted that Mislick and Baldeschwieler’s findings were in the context of a poly-L-lysine mediate transfection of adherent HeLa cells compared to our study which involved cationic-lipid mediated transfection of suspension-adapted HEK293T cells. It is thought that the precise chemistries of the transfection carrier, the properties of the transfection complexes, the cell-type and the chemistries and charge densities of the GAGs all influence transfection performance and may therefore account for these observed differences. The off-target, heparinase-mediated, degradation of cell surface heparan sulphate proteoglycans in addition to the targeted soluble GAGs in the conditioned medium may therefore compromise the ability of lipoplexes to attain efficient transfection. A decision was made to proceed with further investigations focusing exclusively on chondrotinase ABC, since it presented as the most promising candidate for enhanced transfection performance.

### Transfection performance, compromised via the addition of chondroitin sulphate to cultures, is rescued when treating cultures with chondroitinase ABC

To determine whether chondroitinase ABC treatment could reverse the adverse effects of supplementing cultures with chondroitin sulphate on transfection performance, an experiment was conducted which involved the initial supplementation of suspension HEK293T cultures cultivated in 24-DWPs with either 10 µg/mL or 50 µg/mL chondroitin sulphate 4 h prior to transfection. This was then followed by the subsequent addition of chondroitinase ABC 2 h following the chondroitin sulphate addition (i.e., 2 h before transfection).

Compared to untreated controls, the addition of both 10 µg/mL and 50 µg/mL chondroitin sulphate resulted in significant decreases in transfection efficiency (*p* < 0.0001) (Figure 3C), GFP production (*p* < 0.0001) (Figure 3D) and functional LVV titres (*p* < 0.0001) (Figure 3E). Subsequent chondroitinase ABC treatment was able to completely rescue transfection performance when administered at concentrations of 0.0001 U/mL and 0.001 U/mL to cultures that had been previously treated with 10 µg/mL and 50 µg/mL chondroitin sulphate, respectively, with comparable transfection efficiencies (*p* = 0.9648 and *p* = 0.3365, respectively), levels of GFP production (*p* = 0.7233 and *p* = 0.2502, respectively) and functional LVV titres (*p* = 0.9964 and *p* = 0.9461, respectively) being obtained compared to untreated controls (Figures 3C–E). At the higher concentrations of chondroitinase ABC evaluated, enhancements in transfection performance compared to untreated controls were observed despite earlier chondroitin sulphate treatment. A concentration of 0.1 U/mL yielded significant improvements in both transfection efficiency (*p* < 0.0001 and *p* = 0.0005) and GFP production (*p* < 0.0001 and *p* < 0.0001) when added to cultures previously supplemented with 10 µg/mL and 50 µg/mL chondroitin sulphate, respectively. It is therefore postulated that at the higher concentrations explored in the experiment, chondroitinase ABC was more effectively able to degrade both the chondroitin sulphate artificially added to the cultures as well as any existing chondroitin sulphate already present in the conditioned culture medium. Interestingly, the transfection efficiency achieved in the process control differed between Figure 3A (48.6%) and Figure 3C (70.6%), as did the ability of the chondroitinase ABC treatment to yield larger improvements in transfection performance across the range of concentrations investigated. Whilst the increase in transfection efficiency achieved was statistically significant in Figure 3C, the magnitude of the overall increase was lower when compared with Figure 3A. This is likely explained, in part, due to the prior addition of large quantities of chondroitin sulphate prior to chondroitinase treatment and transfection in Figure 3C but it may also indicate that chondroitinase ABC has the ability to yield greater increases in transfection performance when the transfection efficiency is lower to begin with.

### Supplementing HEK293T cultures with chondroitinase ABC enhances transfection performance and LVV production under certain conditions

**FIGURE 4:**
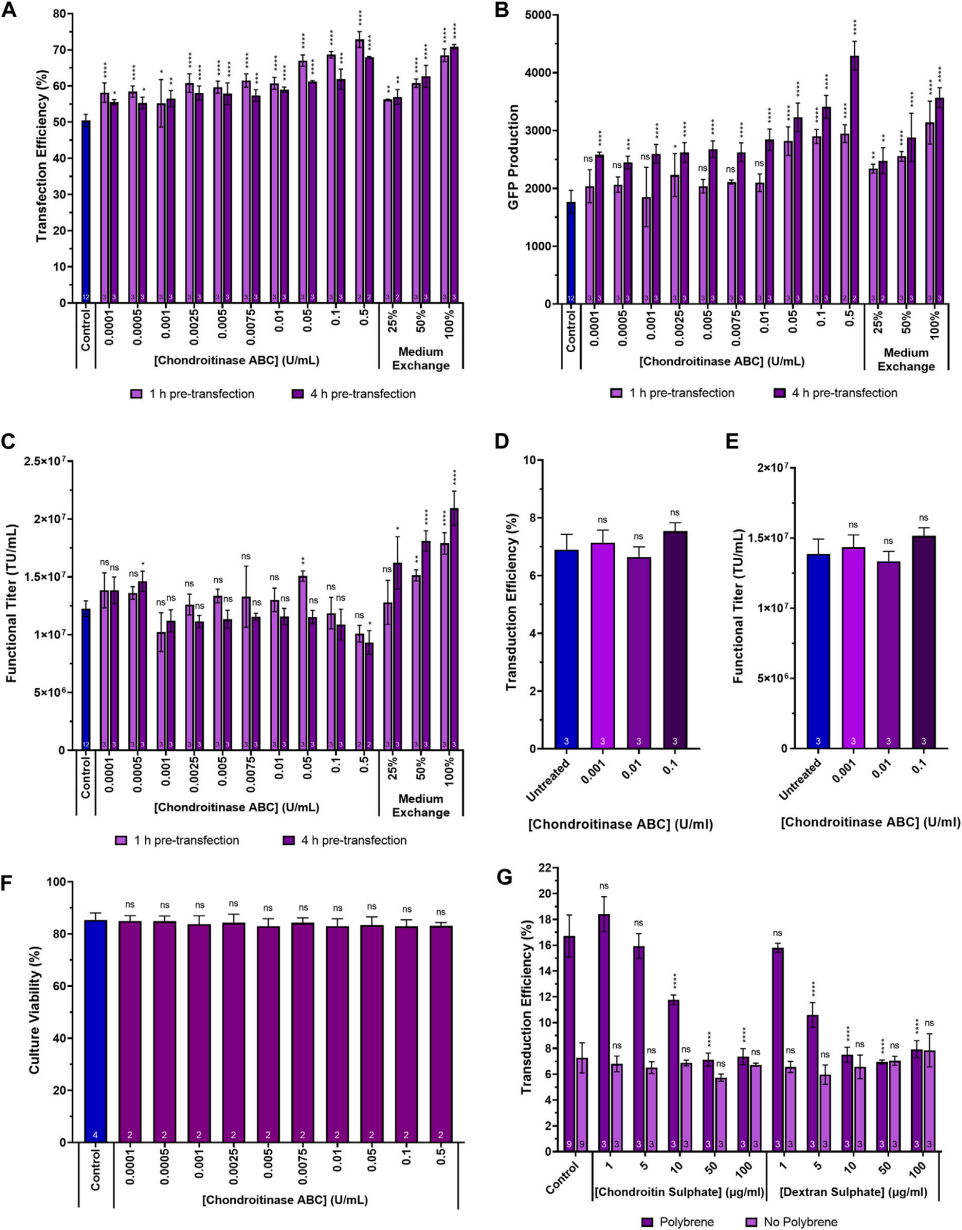
Transfection performance when supplementing cultures with a range of chondroitinase ABC concentrations prior to transfection. **(A)** Transfection efficiency, **(B)** GFP production data measured 24 h post-transfection and **(C)** functional LVV titres achieved following the supplementation of HEK293T cultures with varying concentrations of chondroitinase ABC both 1 h and 4 h prior to transfection. **(D)** Transduction efficiency of adherent HEK293T cells transduced by HIV-1-GFP LVV particles in the presence of chondroitinase ABC, following a 400-fold dilution of the LVV. **(E)** Functional titre calculated for the HIV-1-GFP LVV stock used to transduce adherent cells, in the presence of chondroitinase ABC. **(F)** Cell culture viability at harvest following treatment of cultures with chondroitinase ABC 2 h prior to transfection. **(G)** Transduction efficiency of adherent HEK293T cells transduced by HIV-1-GFP particles following pre-treatment with either chondroitin sulphate or dextran sulphate, in the presence and absence of polybrene. Data points **(A–G)** are the average of replicate cultures with the number of replicates indicated at the bottom of each bar. All error bars are ± one standard deviation of the mean.

To assess the impact of chondroitinase ABC treatment on transfection performance, suspension adapted HEK293T cultures cultivated in 24-DWPs were supplemented with chondroitinase ABC ranging in concentration from 0.0001 U/mL to 0.5 U/mL. Two enzymatic incubation times were investigated, involving the addition of chondroitinase ABC to cultures either 1 h or 4 h prior to transfection. This was done to examine whether a wider range of chondroitinase ABC incubation periods would yield the same enhancement in transfection performance as the 2-h incubation used in the previous experiment. Significant increases in transfection efficiency were detected across the full range of chondroitinase ABC concentrations and incubation times evaluated, with the largest increases being observed at the highest enzyme concentrations investigated (Figure 4A). Increases in transfection efficiency of 22.4% (*p* < 0.0001) and 17.5% (*p* < 0.0001) were observed at the highest concentration of 0.5 U/mL when administered 1 h and 4 h pre-transfection, respectively. Interestingly, the general trend was that larger increases in transfection efficiency were observed when subjecting cultures to chondroitinase ABC treatment for 1 h prior to transfection rather than a 4-h exposure (*p* = 0.0017), but analysis of the GFP production data (Figure 4B) revealed that the opposite was true, where a 4-h exposure resulted in significantly improved levels of GFP expression compared to a 1-h exposure (*p* < 0.0001). The reason for this discrepancy is presently unclear. Whilst increases in GFP production of 66.9% (*p* < 0.0001) and 143.2% (*p* < 0.0001) compared to untreated controls were observed at the highest chondroitinase ABC concentration of 0.5 U/mL when administered 1 h and 4 h pre-transfection, respectively, the anticipated corresponding improvements in functional LVV titre were generally not observed across any of the chondroitinase treatments (Figure 4C). As illustrated in Figure 1, it was initially hypothesised that enhanced transfection performance, achieved via the degradation of accumulated GAGs, would likely lead to increased LVV production. The lack of correlation between these metrics in this experiment indicated that the relationship is more complex.

An alternative method that can be employed to remove chondroitin sulphate and any other inhibitory molecules that may accumulate in conditioned medium over time is to perform a medium replacement/exchange processing step immediately prior to transfection. This involves either the partial or complete removal of the existing conditioned medium and replacement with fresh medium to supply cells with fresh nutrients and to remove inhibitory by-products that may accumulated prior to the transfection step. The fresh medium should be devoid of any inhibitory molecules, including chondroitin sulphate. In suspension culture systems, medium replacement is usually reliant on a centrifugation step, which can be complex to scale-up, but can also be mediated by perfusion technologies including hollow fibres and acoustic cell retention devices (Ansorge et al., 2009). The chondroitinase ABC treatment method was directly compared to a medium replacement strategy which involved the centrifugation-mediated replacement of either 25%, 50% or 100% of the conditioned medium with fresh FreeStyle 293 Expression Medium, performed either 1 h or 4 h prior to transfection. All media replacement strategies yielded significant increases in transfection efficiency, levels of transgene expression and functional LVV titres (with the exception of the 25% medium replacement step when performed 1 h pre-transfection) (Figure 4). Larger improvements in titre were obtained when the step was performed 4 h prior to transfection compared to 1 h prior (*p* = 0.0019). This is potentially attributable to the decrease in culture viability that is associated with the centrifuge-mediated medium exchange, where cultures had a longer period of time to recover when the step was performed 4 h prior to transfection. Interestingly, the maximum transfection efficiencies and levels of GFP transgene expression were comparable between the 0.5 U/mL chondroitinase ABC treatment and the 100% medium replacement strategy which would suggest that chondroitin sulphate accounts for the majority of the inhibitory activity of the conditioned medium on transfection performance compared to other inhibitory molecules that may accumulate over time. Albeit in a different context, similar observations were previously made by Le Doux and colleagues when investigating the impact of proteoglycan removal on retroviral transduction; it was reported that up to 76% of the inhibitory activity of the conditioned cell culture medium was sensitive to chondroitinase ABC treatment (Le Doux et al., 1998).

The addition of chondroitinase ABC to HEK293T suspension cultures, in the context of a control LVV production process, failed to yield improved LVV titres (Figure 4C). To rule out the possibility that residual chondroitinase ABC in viral vector harvest samples was adversely interfering with the transduction assay and masking any improvements in functional titre, adherent HEK293T cell populations were exposed to HIV-1-GFP LVV particles in the presence of three concentrations of chondroitinase ABC. There was no significant difference in the transduction efficiency of the adherent cells treated with chondroitinase ABC and the untreated control (Figure 4D); titres calculated from the transduction efficiencies, using Eq. 3, were equivalent between the conditions (Figure 4E). To determine whether a compromised culture viability, following a prolonged exposure of cells to chondroitinase ABC, was accountable for the absence of an improvement in LVV production, culture viabilities were assessed at the end of the process following the pre-transfection treatment of cultures with a range of chondroitinase ABC concentrations (0.0001 U/mL–0.5 U/mL). Culture viability was found to be comparable in all conditions (Figure 4F).

Soluble polyanions including heparin, dextran sulphate and heparan sulphate have been shown to prevent the attachment of HIV-1 virions to host cells (Ito et al., 1987; Baba et al., 1988; Mitsuya et al., 1988; Bobardt et al., 2004). We hypothesised that the digestion of chondroitin sulphate-based GAGs to enhance transfection may be removing a potential barrier to viral vector auto-transduction. Auto-transduction describes a process whereby viral vector producing cells are transduced by newly synthesised lentiviral vector particles produced during the LVV production process; this is an undesirable process inefficiency resulting in the loss of vector particles (Elizalde and Ramírez, 2021). If the digestion of chondroitin sulphate resulted in enhanced viral vector loss, then this may explain the lack of improvement in titre despite significant improvements in transfection performance. To test whether differences in the concentration of chondroitin sulphate affected transduction efficiency, adherent HEK293T cell cultures were treated with varying concentrations (1 µg/mL–100 µg/mL) of either chondroitin sulphate or the GAG-analogue, dextran sulphate, in 12-well plates prior to exposing cells to HIV-1-GFP LVV particles (Figure 4G). The experiment was carried out both in the presence and absence of the transduction enhancer, polybrene, which was added to achieve a final concentration of 8 µg/mL when present. Chondroitin sulphate and dextran sulphate supplementation did not reduce transduction efficiencies in the absence of polybrene, even at the highest concentration evaluated. The only apparent inhibitory effect that GAG and GAG-analogue supplementation had on transduction efficiency was to neutralise the transduction enhancement effect of polybrene; this was achieved at a concentration of 50 µg/mL chondroitin sulphate and 10 µg/mL of dextran sulphate where, at these concentrations, polybrene treated cells yielded comparable transduction efficiencies to untreated cells. The difference is likely due to the increased sulphation, and resulting higher negative charge density, of dextran sulphate relative to chondroitin sulphate allowing it to neutralise the positive impact of polybrene more efficiently. However, LVV production processes occur in the absence of polybrene and the lack of any significant transduction inhibition in the chondroitin sulphate supplemented conditions (in the absence of polybrene) suggest that digestion of chondroitin sulphate via chondroitinase ABC treatment was not enhancing rates of vector loss via auto-transduction during LVV production. It is possible that chondroitinase treatment, whilst enhancing transfection, may potentially be having an impact on the subsequent steps of viral vector assembly and budding which may be masking the anticipated improvement in titre.

Another potential explanation for the lack of improvement in titre following chondroitinase treatment was that the control process, which had been highly optimised, ensured the addition of a sufficiently large number of lipoplexes during the transfection step to satisfactorily overcome the GAG barrier to transfection and ensure an adequate level of gene delivery. It was hypothesised that in situations where the concentration of chondroitin sulphate may be higher prior to transfection, for example, due to an increased cell concentration prior to transfection (Figure 2C), then chondroitinase treatment-mediated improvements in titre would be more likely observed. To test this hypothesis, cells were inoculated in 24-DWPs at double the cell density compared to the control process which ensured approximately double the number of cells (4 × 10^6^ viable cells/mL) prior to transfection, compared to the control.

**FIGURE 5:**
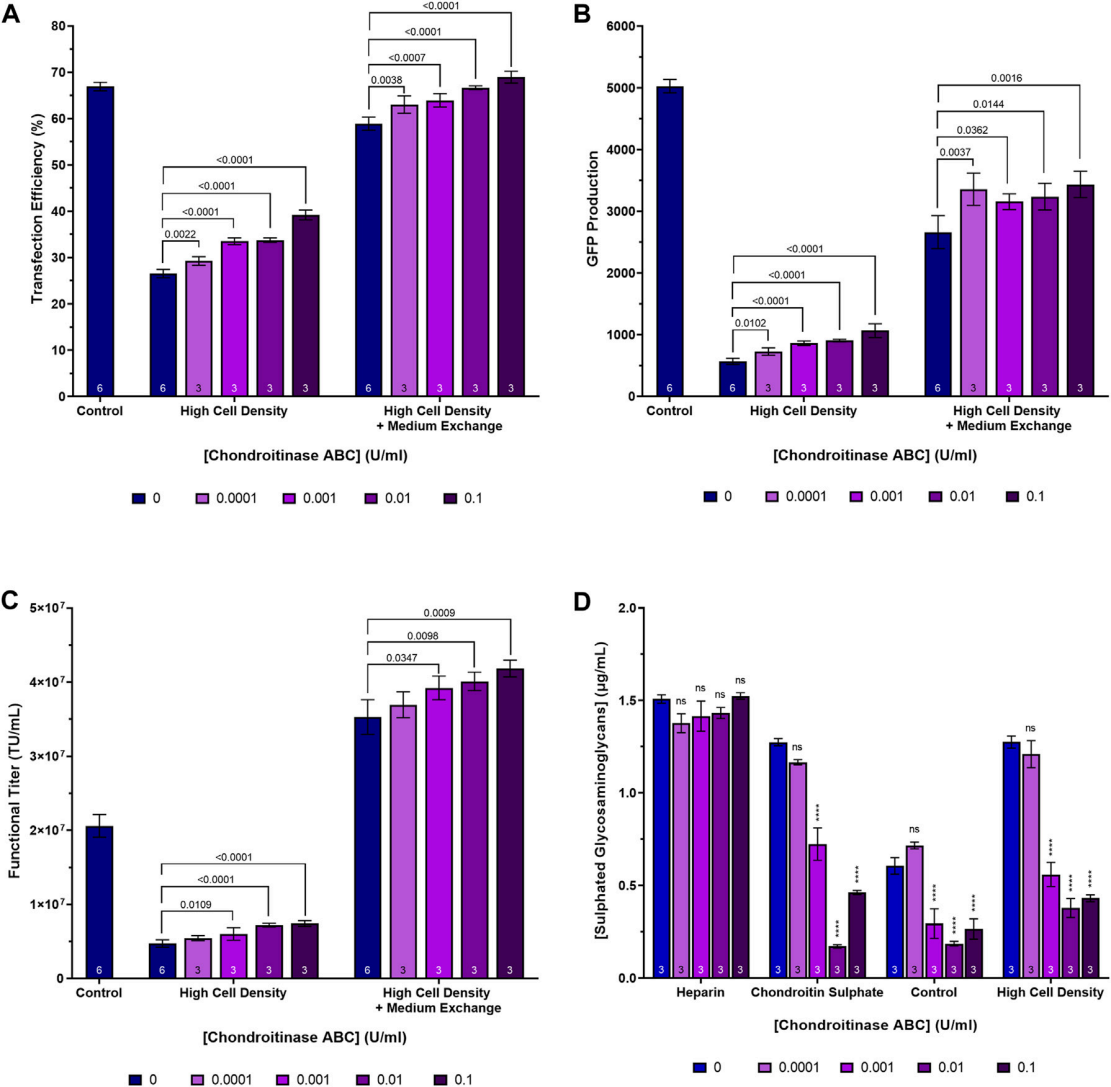
Transfection performance when supplementing cultures cultivated at high cell densities with a range of chondroitinase ABC concentrations prior to transfection. **(A)** Transfection efficiency, **(B)** GFP production data measured 24 h post-transfection, **(C)** functional LVV titres, and **(D)** extracellular sulphated glycosaminoglycan concentrations achieved following the supplementation of heparin and chondroitin sulphate stock solutions and HEK293T cultures with varying concentrations of chondroitinase ABC, 2 h prior to transfection. “Control” in **(A–C)** refers to a standard cell density LVV production process which does not involve any pre-transfection treatment of cells with chondroitinase ABC. Data points **(A–D)** are the average of replicate cultures with the number of replicates indicated at the bottom of each bar. All error bars are ± one standard deviation of the mean.

In the case of the high cell density process, two versions were executed, one that involved the performance of a post-transfection, centrifugation-mediated, medium exchange 3 h following transfection and one that did not. The purpose of the post-transfection medium exchange was to ensure the provision of sufficient nutrients to the cells to ensure that they were able to remain viable throughout the duration of the process. Cultures were supplemented with chondroitinase ABC at four concentrations ranging from 0.0001 U/mL to 0.1 U/mL, 2 h prior to transfection. The highest concentration of 0.5 U/mL utilised in the previous experiments was excluded from this experiment as, sufficiently large improvements in transfection performance were achieved at a concentration of 0.1 U/mL and there were concerns that the high cost of the enzyme would prohibit its use at a concentration of 0.5 U/mL in future scale-up experiments. Enzymatic treatment of the high cell density cultures resulted in a 66.2% reduction in the concentration of sulphated glycosaminoglycans (*p* < 0.0001) when chondroitinase ABC was used at a concentration of 0.1 U/mL, suggesting that the majority of the sulphated glycosaminoglycans present in the conditioned medium are chondroitin sulphate (Figure 5D). In addition to reducing the extracellular concentration of glycosaminoglycans, enzymatic treatment of the high cell density cultures with chondroitinase ABC yielded significant, dose-dependent, increases in transfection efficiency and GFP production at all concentrations tested (Figures 5A, B). Crucially, chondroitinase ABC was found to result in a significant increase in functional LVV titre under these conditions, with a concentration of 0.1 U/mL resulting in a 57.6% (*p* < 0.0001) and an 18.6% (*p* = 0.0009) increase in titre for the high cell density cultures not subjected to a medium exchange and those that were, respectively, compared to the respective high cell density culture controls (Figure 5C). When used in combination, doubling the initial inoculation cell density, treating cells with 0.1 U/mL chondroitinase ABC prior to transfection and performing a medium exchange 3 h post-transfection, resulted in a 2.0-fold increase in functional LVV titre compared to the control process. Based on an average final cell concentration of 5.69 × 10^6^ viable cells/mL in this condition at harvest, an average of 7.4 TU/cell were produced. This was comparable to the average number of functional particles produced per cell in the control (7.2 TU/cell) where a final cell concentration of 2.86 × 10^6^ viable cells/mL was measured at harvest. As anticipated, a post-transfection medium exchange was required in the high cell density process to ensure that cells remained productive for the duration of the process. The high cell density process that was not subjected to a post-transfection medium exchange resulted in reduced transfection efficiencies, levels of transgene expression and titres compared to the standard cell density control, likely due to nutrient exhaustion in the crucial LVV production phase. It is anticipated that the improved performance attained in the high cell density process which employed the post-transfection medium exchange could likely be achieved through the use of concentrated nutrient feeds as an alternative to a medium exchange protocol which are more complex and costly to scale up. Additional experiments would need to be conducted to verify this.

The addition of chondroitinase ABC to cultures presents a simple and scalable processing step that we anticipate would be applicable for large scale transient transfection-based bioprocesses. Calculations would need to be carried out on a process-by-process basis to determine the value of performing such a step at large scale versus the cost of raw materials to determine the viability of such an approach. Residual enzyme present at the end of the upstream LVV production process would likely be easily removed in standard tangential flow filtration unit operations that are typically employed in viral vector downstream purification processes; indeed such methods are already employed for the removal of Benzonase^®^, a promiscuous endonuclease frequently used in viral vector bioprocesses to ensure sufficient removal of contaminating nucleic acids, to ensure compliance with regulatory purity standards for biopharmaceuticals (Merten et al., 2014). It is recognised that the addition of enzymes, such as Benzonase^®^, is an expensive requirement in large-scale viral vector manufacturing processes and alternative approaches are being investigated. One group has engineered an inducible HEK293T cell line with the ability to secrete a *Staphylococcus aureus* nuclease during LVV production to reduce DNA impurity levels with the ultimate goal of omitting costly Benzonase^®^ additions (Ali et al., 2023; Howe et al., 2024). A similar approach could be taken to digest chondroitin sulphate-based GAGs prior to transfection through the generation of a cell line engineered to secrete chondroitinase ABC, avoiding costly additions of enzyme to bioreactors in the context of larger scale LVV production.

## Conclusion

Transfection-based bioprocesses are highly sensitive and dependent on a large number of intrinsic factors related to the properties of lipoplex and polyplex-based transfection complexes and extrinsic factors related to cell culture conditions (van Gaal et al., 2011). In this work we have demonstrated that transfection performance can be significantly enhanced via the enzymatic digestion of specific extracellular chondroitin sulphate-based glycosaminoglycans that are present in conditioned cell culture medium prior to transfection. We were able to demonstrate up to a 22.4% increase in transfection efficiency and a 2.4-fold increase in GFP expression when treating cultures with 0.5 U/mL chondroitinase ABC prior to transfection. Whilst the anticipated corresponding increase in functional LVV titre was not observed under the operating conditions employed as part of the control LVV production process, significant improvements in titre were observed when cells were cultivated at higher cell densities that those utilised in the established process. A 71.2% increase in functional LVV titre was calculated when doubling the cell density at the point of transfection compared to the platform process and a further 18.6% (*p* = 0.0009) increase in titre was calculated when supplementing the high-density cell cultures with 0.1 U/mL chondroitinase ABC prior to transfection. Whilst this work specifically sought to increase transfection efficiencies in the context of a lentiviral vector production bioprocesses, we anticipate that the improved transfection efficiencies that we report in this context will likely be observed in a wide range of transient gene expression-based systems, including those used for the transient production of other biologics.

Medium replacement was explored as an alternative strategy to the targeted enzymatic degradation approach for reducing the concentration of inhibitory molecules in the conditioned medium. In suspension culture systems, medium exchanges are usually reliant on either centrifugation steps or perfusion technologies, including hollow fibres and acoustic cell retention devices (Ansorge et al., 2009). Indeed, such technologies, including tangential flow depth filtration, have already been used by us and others to mediate larger scale medium exchanges and perfusion in the context of lentiviral vector production processes (Williams et al., 2020; Tran and Kamen, 2022). These approaches enable the deployment of a global medium replacement strategy involving the removal of all inhibitory molecules that accumulate in the conditioned cell culture medium prior to transfection, rather than the targeted degradation of specific inhibitory molecules that have been identified as being problematic for transfection-based bioprocesses. Whilst this less specific approach may present a more desirable solution for certain large-scale bioprocesses, the targeted degradation of chondroitin sulphate via chondroitinase ABC would likely be more applicable to batch or fed batch-based biomanufacturing processes where improvements in transfection performance and productivity may be made.

## Data Availability

The raw data supporting the conclusion of this article will be made available by the authors, without undue reservation.

## Abbreviations

CHO, Chinese hamster ovary; DWP, deep well plate; FDA, Food and Drug Administration; GAG, glycosaminoglycan; GFP, green fluorescent protein; HEK, human embryonic kidney; HIV-1, human immunodeficiency virus type-1; LVV, lentiviral vector; MFI, median fluorescence intensity; TU, transducing units; VSV-G, vesicular stomatitis virus G envelope protein.
