# Supplementary material for: Degradation of specific glycosaminoglycans improves transfection efficiency and vector production in transient lentiviral vector manufacturing processes
Source: Front Bioeng Biotechnol. 2024 Jun 26;12:1409203. doi: 10.3389/fbioe.2024.1409203 (PMC11238175; doi:10.3389/fbioe.2024.1409203)
Supplement: Supplementary file 1 [file DataSheet1.docx]

**Figure S1:** Gating strategy for identifying single, live, GFP positive cells within populations that were either un-transfected or transfected with plasmids required to produce a HIV-1-GFP LVV. Cell populations were either untreated or treated with 0.5 U/mL chondroitinase ABC one hour prior to transfection. Cell samples were taken and analysed via flow cytometry 24 hours post-transfection. Gate 1: forward scatter area (FSC-A) versus side scatter area (SSC-A) gate to identify the main cell population of interest, excluding debris, dead cells and cell aggregates. Gate 2: FSC-A versus forward scatter height (FSC-H) gate to refine the single live cell population. Gate 3: FSC-A versus GFP gate to identify GFP positive cells within the single live cell population.
